# Feasibility and outcome of the fewer falls in multiple sclerosis intervention: a pilot randomized controlled trial

**DOI:** 10.1038/s41598-025-27071-0

**Published:** 2025-11-18

**Authors:** Ulrika Meijer, Charlotte Ytterberg, Kristina Gottberg, Fredrik Piehl, Maria Flink, Marie Kierkegaard

**Affiliations:** 1 Department of Neurobiology, Care Sciences and Society, Karolinska Institutet, Huddinge, Sweden; 2 Women’s Health and Allied Health Professionals Theme, Karolinska University Hospital, Stockholm, Sweden; 3 Department of Clinical Neuroscience, Karolinska Institutet, Stockholm, Sweden; 4 Academic Specialist Center, Center of Neurology, Stockholm Health Services, Stockholm, Sweden

**Keywords:** Neurological rehabilitation, Fall prevention, Self-management, Digital health, Diseases, Health care, Medical research, Neurology, Neuroscience

## Abstract

Falls are common among people with multiple sclerosis (PwMS) and bring risk of both injury and reduced quality of life. The multifactorial background to risk of falls necessitates adapted and comprehensive interventions. The “*Fewer Falls in MS”* intervention is a manualized online self-management fall prevention programme designed for both ambulatory and non-ambulatory PwMS. We conducted a pilot trial to evaluate feasibility and outcome of the *Fewer Falls in MS* to determine whether to advance to a full-scale randomized controlled trial (RCT). In this two-armed parallel group study, 46 adult PwMS from across Sweden were randomized to the intervention (n = 23) or control (n = 23) group. The intervention included six 2-hour weekly online group sessions led by a trained group leader, followed by a booster session 8 weeks later. Both groups received a brochure on fall prevention. Falls were monitored weekly for 18 weeks via SMS. The evaluation demonstrates that participant recruitment and retention, data collection, intervention delivery, session adherence, and outcome measures were feasible. At the 18 weeks follow-up, results from the standardized questionnaires and falls data revealed no significant between-group differences. Furthermore, no adverse events were reported. Meeting all, but one, progression criteria justify proceeding to a full-scale RCT, with insights from this pilot trial being used to further refine the intervention and outcome measures.

**Supplementary Information:**

The online version contains supplementary material available at 10.1038/s41598-025-27071-0.

## Introduction

Multiple sclerosis (MS) is a chronic, inflammatory, demyelinating, and neurodegenerative disease of the central nervous system, with a usual onset between 20 and 40 years of age. The prevalence has increased over the last decade and is globally 36 per 100,000 population, meaning that about 2.8 million people live with MS worldwide^1^. The disease is typically relapsing-remitting at onset, but later converting to a progressive worsening, where clinical symptoms depend on the extent and distribution of demyelinating lesions throughout the central nervous system. Overall, MS has the potential to impact a wide variety of body-function impairments, activity limitations and participation restrictions^2^, which increase the risk for a fall, i.e., “an unexpected event in which the participants come to rest on the ground, floor, or lower level"^3^.

Existing literature indicates that a majority of people with MS (PwMS) fall at least once within periods of three to six months^4–6^. For PwMS, both individual factors and the interaction between various environmental and behavioural factors contribute to risk of falls^4,6–10^. Falls occur in both PwMS who are ambulatory with mild disability^11^ and in those who are non-ambulatory^9^ i.e., those capable of walking only a few steps or not at all. Further, almost 40% of PwMS are frequent fallers i.e., ≥ 2 falls^5,12^, and between 30% and 50% of those who fall report injurious falls^4,6^. While most reported fall-related injuries in PwMS are minor, such as bruises that do not require medical attention^13,14^, MS is associated with an increased risk of fractures^15^. Notably, non-ambulatory PwMS with poor transfer ability are particularly vulnerable to more serious fall-related injuries^16^.

Falls are of major concern for both PwMS and for the society not only due to risk of injury, but also that fear of falling restricts mobility and lowers quality of life^17,18^. Hence, effective fall prevention interventions are needed. Historically, fall prevention interventions have mainly focused on physical impairments, such as compromised balance, and have not addressed the diverse and interacting factors influencing the risk of falls^19,20^. Further, a significant gap in current research is the lack of consideration of behavioural influences on fall risk. In addition, few interventions are tailored for the non-ambulatory MS population^21,22^.

In chronic diseases such as MS, the variable individual presentation of risks of fall may be reduced through self-management. Self-management can be defined as “the individual’s ability to manage the symptoms, treatment, physical and psychosocial consequences, and lifestyle changes intrinsic in living with chronic conditions”^23^. To our knowledge, the published MS fall prevention studies that include some self-management features are three studies with a randomized controlled trial (RCT) design^24–26^, one feasibility RCT^27^, two pilot RCTs^28,29^, and three pilot studies with a pre/postintervention design^30–32^. Identified shortcomings with these interventions are the lack of self-management definition; none included both ambulatory and non-ambulatory PwMS; and that few, if any, were developed in co-creation with PwMS. Consequently, as shown in our scoping review^33^, there is a need of additional research to address feasibility and effectiveness of self-management fall prevention interventions in PwMS.

Thus, together with PwMS and healthcare professionals, we co-created the online self-management fall prevention intervention *Fewer Falls in MS*, as described in recent publications^34,35^. This intervention is novel in several ways: it is developed in close collaboration with stakeholders; it targets both ambulatory and non-ambulatory PwMS; it focuses on self-management; and it is delivered online. By including participants with a wide range of disability levels, the intervention aims to be inclusive and relevant to a broader MS population. However, this diversity may introduce challenges in tailoring content and interpreting outcomes across subgroups. Therefore, it is particularly important to assess the feasibility of the intervention before progressing to a full-scale trial. Consequently, a pilot RCT with a mixed-methods design, as outlined in our study protocol^36^, was performed in accordance with the Medical Research Council framework on complex interventions^37^. We report here results on feasibility and outcome of the intervention, adhering to the CONSORT statement extension for pilot trials^38^. Results on acceptability and fidelity will be reported in a separate qualitative paper.

## Objectives

The overall aim was to evaluate the feasibility and outcome of the *Fewer Falls in MS*, to inform the decision on whether to proceed to a full-scale RCT (see Table 1 for progression criteria). The specific objectives were to evaluate:

**Table 1:** Progression criteria.

	Progression criteria	Results	Criteria met
Recruitment process	Recruit at least 75% of the target sample size (48 participants) within 3 months	46 participants (96%) were recruited within 46 days	Yes
Data collection procedures	At least 80% of participants complete the baseline data assessments within 60 min. At least 80% of participants complete all data collection (T0, T1, T2, and SMS)	All participants completed the baseline assessment within 60 min. The overall response rate exceeded 80% (T0 = 100%, T1 = 98%, T2 = 96%, SMS = 97%)	Yes
Intervention delivery	At least 80% of sessions completed within the assigned time frame and without major technical issues	19 (90%) sessions were completed within the assigned time frame	Yes
		Minor technical issues during first three sessions	Partly
Outcome measures	At least 80% strongly agree on the relevance of the outcome measures	27 (61%) strongly agreed	No
	No floor or ceiling effects in standardized questionnaires	No floor or ceiling effects were found	Yes
Participant retention	At least 80% retention rate. Number and reasons for dropouts	42 of 46 (91%) participants remained in the trial and participated in all data collection. One participant dropped out for personal reasons	Yes
Session adherence	At least 80% complete 5 of 7 sessions	18 of 20 (90%) participants who started the intervention attended at least 5 sessions	Yes

1. Feasibility of the recruitment process.
2. Feasibility of data collection procedures.
3. Feasibility of delivery of the intervention.
4. Feasibility of outcome measures.
5. Participant retention.
6. Session adherence.
7. Outcome of the intervention (between- and within-group differences).
8. Adverse events.

## Methods

### Trial design

A two-armed parallel group pilot RCT was performed with a 1:1 ratio. Full details of the methodology are provided in the study protocol^36^.

### Participants and recruitment

Eligible participants were ambulatory and non-ambulatory PwMS aged ≥ 18 years, who were able to understand and communicate in Swedish; had access to and the self-rated ability to use technical devices for online meetings, such as computers, tablets, or smartphones with internet access; and capable of giving informed consent. Non-ambulatory PwMS were those unable to walk beyond 5 m even with aid, who could independently transfer from bed to wheelchair, with or without aids. A sample size of 48 participants was pragmatically decided based on having three online intervention groups, each consisting of a maximum of eight participants and led by one of three trained group leaders. This sample size was also deemed sufficient for accurately estimating recruitment and retention rates^39,40^.

Recruitment and screening took place between December 2021 and February 2022. Participants were recruited from two outpatient neurology clinics in Stockholm, Sweden, and through the patient organization Neuro Sweden. Information about the pilot trial and contact details were available at the outpatient clinics, on one of the clinic’s website, and in Neuro Sweden’s digital newsletters and social media. Interested participants contacted a research assistant (UM) via email or phone. The research assistant provided additional information and screened participants for eligibility over the phone. Written information was sent to eligible interested PwMS. Those who returned a signed consent were emailed a link to the online baseline assessment via Research Electronic Data Capture (REDCap), a secure, web-based solution for collecting and managing research data.

**Fig. 1:**
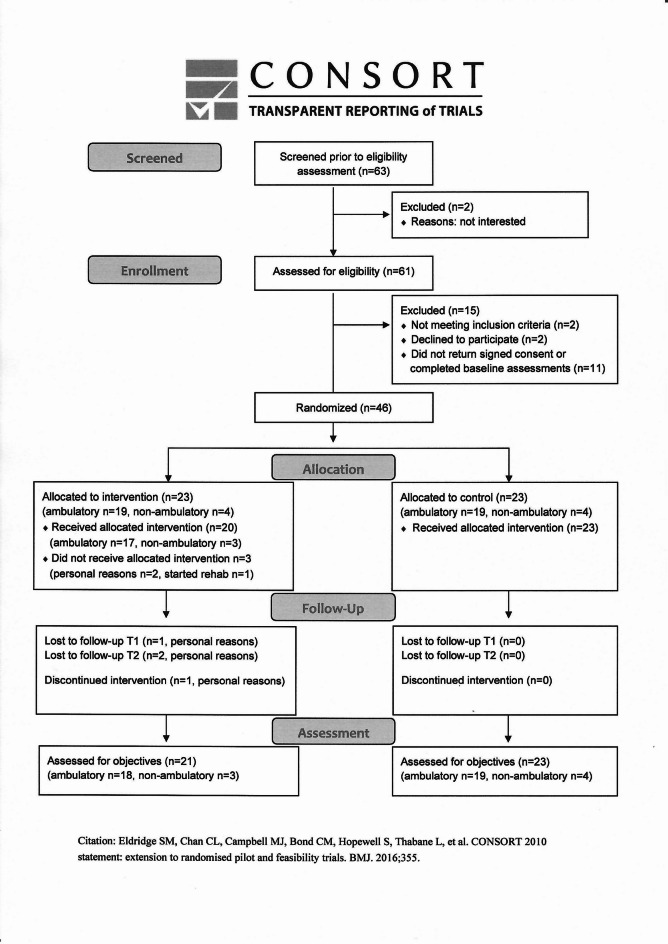
CONSORT flow diagram.

A member of the research group (MK) used a computer-generated random scheme (Sealed Envelope Ltd) for randomization with a 1:1 allocation ratio with blocks of four, stratified by ambulation level (ambulatory/non-ambulatory). Randomization was performed using sequentially numbered, sealed, opaque envelopes. A research assistant (UM) assigned participants after baseline assessments to intervention group (IG) or control group (CG). Figure 1 (CONSORT flow diagram) shows participants’ flow through the trial. Baseline data are described in Table 2.

**Table 2:** Baseline data for intervention group (*n* = 23) and control group (*n* = 23) participants.

	Intervention group	Control group
Age, years, mean (range)	58 (43–72)	57 (39–72)
Women, n (%)	19 (83)	18 (78)
*Education level*		
University, n (%)	13 (57)	19 (83)
Secondary school, n (%)	10 (43)	4 (17)
Working full or part time, n (%)	13 (57)	11 (48)
Years since diagnosis, mean (range)	15 (1–39)	15 (2–41)
*Expanded disability status scale (EDSS)*		
Mild (EDSS 0-3.5), n (%)	6 (26)	6 (26)
Moderate (EDSS 4-6.5), n (%)	13 (57)	13 (57)
Severe (EDSS ≥ 7), n (%)	4 (17)	4 (17)
Fatigue, yes, n (%)	19 (83)	17 (74)
*Self-reported falls data previous 3 months*		
Number of fallers, n (%)	12 (52)	16 (70)
Frequent fallers (≥ 2 falls), n (%)	7 (30)	10 (43)
*Use of indoor mobility aids, n (%)*		
Always, n (%)	7 (30)	4 (17)
Sometimes, n (%)	1 (4)	2 (9)
*Use of outdoor mobility aids, n (%)*		
Always, n (%)	13 (57)	9 (39)
Sometimes, n (%)	2 (9)	5 (22)

Neither participants, group leaders, research assistants, nor the persons performing the data analyses were blinded after randomization, as blinding was incompatible with the intervention.

### The intervention

The *Fewer Falls in MS* is a manualized self-management fall prevention programme based on Social Cognitive Theory^23^ and Universal Design for Learning^41^. The intervention was delivered in three online groups each led by a group leader, a licensed healthcare professionals with expertise in neurological care, rehabilitation, and/or fall prevention. Before initiating the intervention, two researchers (MF, CY) trained the three group leaders in three 2-hour online educational sessions comprising a programme overview and role play. Intervention group participants (IGPs) received six 2-hour weekly group sessions, followed by a booster session eight weeks later. The sessions focused on building IGPs’ self-management strategies through interactive lectures and peer learning. The booster session was of similar duration and was designed to reinforce and expand upon the content covered in initial sessions. The online sessions were delivered in real-time, face-to-face via the Zoom video platform. Sessions 1–6 were conducted from March to April 2022, and the booster session occurred in June 2022. An online learning platform (Canvas, Instructure) was used to distribute intervention materials, including assignments for participants, and for chat communication among participants outside the sessions. The assignments aimed to enable IGPs to develop and use individual action plans for managing fall risks between sessions. Both IGPs and control group participants (CGPs) received a brief informational brochure via email. The brochure included information about fall risk in relation to MS, along with practical tips on how to reduce fall risk in everyday life. The content was designed to be accessible and relevant for individuals with varying levels of disability.

### Data collection

**Fig. 2:**
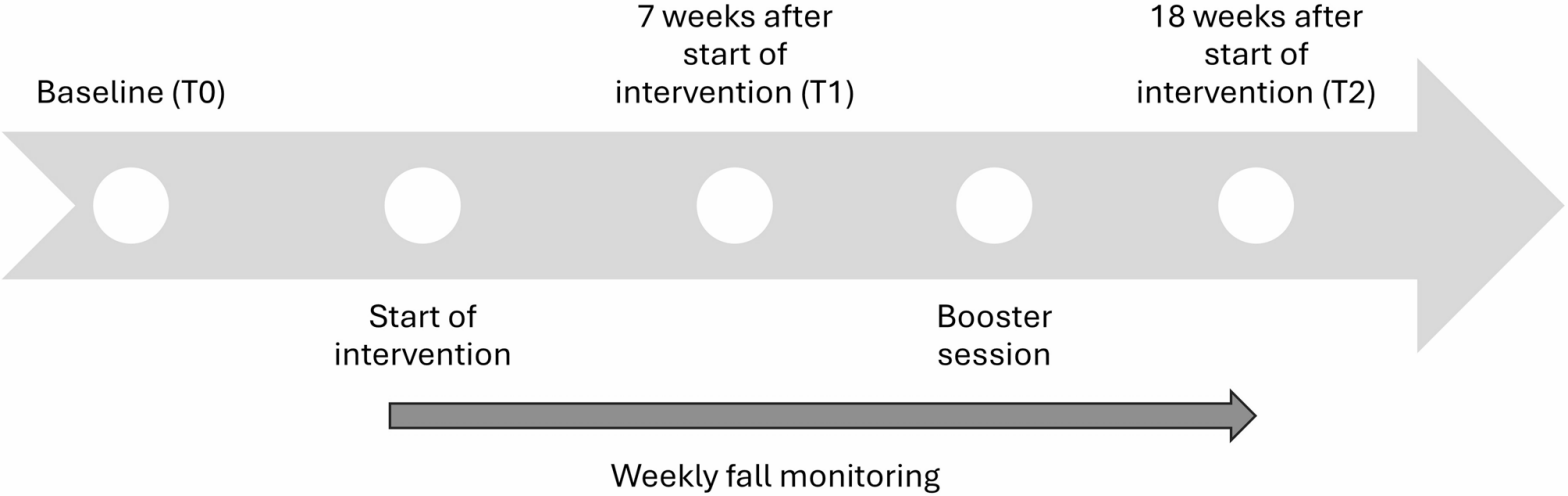
Data collection timeline.

Questionnaire data were collected at three timepoints (Fig. 2) using REDCap online surveys: at baseline (T0), seven weeks after the start of the intervention (T1) i.e., at the end of the Fewer Falls in MS program but before the booster session, and 18 weeks after the start of intervention (T2). The REDCap system was configured to prevent submission of incomplete forms. At baseline (T0), self-reported information on sociodemographic and disease-related characteristics (years since diagnosis, EDSS level based on walking ability, fatigue, use of mobility aids) and data on falls in the previous three months were collected. At T0, T1, and T2, standardized questionnaires were administered to assess fall prevention behaviours, fear of falling, social and everyday activities, perceived impact of MS, physical activity, and sedentary behaviours. Weekly SMS were used to monitor falls throughout the intervention period. In addition, at T2 a questionnaire (Appendix 1) was used to evaluate the participants’ experiences of data collection procedures and measures.

#### Standardized questionnaires

*The Fall Prevention Strategies Survey (FPSS)* A self-report questionnaire comprising 11 items on fall preventive behaviours^42^. Participants indicate the frequency of each behaviour (never, sometimes, regularly). The total score ranges from 0 to 22, with higher scores reflecting greater use of fall preventive strategies. Rasch analysis supports the use of the FPSS to examine the frequency of engaging in protective behaviours related to fall risk among adult PwMS^43^.

*The Visual Analogue Scale for Fear of Falling (VAS-FoF)* A self-report measure of perceived fear of falling^44^, rated on a scale from 0 (no fear) to 100 (worst imaginable fear). The VAS-FoF has shown a fair test–retest reliability and it has been validated for older adults after a fall, but not specifically for PwMS. Participants rated their fear of falling both indoors and outdoors.

*The Falls Efficacy Scale International (FES-I)* A self-report 16-item questionnaire assessing concern about falling during various activities^45^. Responses are given on a four-point scale from 1 (not at all concerned) to 4 (very concerned). The total score ranges from 16 to 64. The FES-I has been validated and reliability tested for ambulatory PwMS^46^. The scale was used for ambulatory participants.

*The Spinal Cord Injury Falls Concern Scale (SCI-FCS)* A self-report 16-item questionnaire developed and validated for non-ambulatory individuals with spinal cord injuries, assessing concern about falling during various activities^47^. Responses are rated from 1 (not at all concerned) to 4 (very concerned). The total score ranges from 16 to 64. It was used for the non-ambulatory participants.

*The Frenchay Activities Index (FAI)* A self-report 15-item questionnaire designed to measure the frequency of instrumental activities of daily living, initially developed and validated for stroke survivors^48^. Activities are rated based on their frequency over the past 3 or 6 months, with scores ranging from 0 (lowest activity level) to 3 (highest activity level). The total score ranges from 0 to 45. FAI has been frequently used in the MS population.

*The Multiple Sclerosis Impact Scale (MSIS-29)* A self-report 29-item valid and reliable questionnaire specifically developed to assess the physical and psychological impact of MS^49,50^. Responses are compiled separately for physical (20 items) and psychological (9 items) aspects and converted to a 0-100 scale (0 representing no impact).

*The Physical Activity Questionnaire* Consists of two questions regarding aerobic physical activity during a typical week^51^. Responses are compiled to generate a total amount of physical activity. The questionnaire has been validated for adults in a general Swedish population^51^. The cut-off for insufficiently physically activity set to < 150 activity minutes/week.

*The Sedentary Behaviour Questionnaire* Consists of one question on time spent sitting on a typical day, excluding sleep^52^. Responses are given using seven categorical options (never to virtually all day). It has been validated for the general population, middle-aged adults and for the elderly, and has shown excellent test-retest reliability. The cut-off for sedentary behaviour was set to ≥ 10 h/day.

#### Fall monitoring

To monitor falls throughout the 18-week intervention period, all participants received an automatically distributed SMS every Saturday via a web-based service (iP.1 Networks AB), asking whether they had experienced a fall during the past week (yes/no). Participants who responded ‘yes’ were contacted by telephone the following week by one of two research assistants with follow-up questions regarding the number of falls, circumstances, perceived causes, and any consequences including fall-related injuries. The responses were manually entered into the REDCap data collection system. All reported injurious falls were categorised as minor, moderate, or serious injuries^53^. Before the start of fall monitoring, participants were informed via email that a fall is “an unexpected event in which the participants come to rest on the ground, floor, or lower level”^3^.

#### Observations

Members of the research team (CY, MF, UM) observed each online session in the *Fewer Falls in MS*. They took notes using a structured observation protocol regarding the feasibility of the delivery of the intervention.

### Assessment of objectives

1. The feasibility of the recruitment process was evaluated based on the time needed to complete participant recruitment. This evaluation included the number of interested and eligible PwMS; the number of eligible PwMS who agreed to participate; and the time needed for the screening process.
2. The feasibility of data collection procedures was evaluated through the time needed to complete baseline assessment (T0), and number of participants who completed T0, T1, and T2 assessments. In addition, the feasibility of using SMS to collect data on falls was evaluated based on the response rate. The feasibility of using telephone follow-ups to collect data on falls was evaluated based on the number of follow-up calls and the approximate time required for each call. Furthermore, participants’ experiences of data collection procedures were evaluated through an online questionnaire administered at T2 (Appendix 1).
3. The feasibility of delivery of the intervention was evaluated through number of sessions completed within the assigned time frame and occurrence of technical issues.
4. The feasibility of outcome measures was evaluated based on participants’ experiences of measures at T2 (Appendix 1). In addition, feasibility of the standardized questionnaires was evaluated through assessment of floor and ceiling effects, defined as ≥ 15% of participants scoring the minimum and maximum possible score, respectively^54^.
5. Participant retention was evaluated by the number of participants who remained in the trial, number of dropouts and reasons.
6. Session adherence was evaluated by the number of IGPs attending the online sessions.
7. Outcome of the intervention was evaluated by analyses of between- and within-group differences regarding falls data and standardized questionnaires.
8. Adverse events were defined as any fall-related injuries that IGPs perceived to be related to activities in their action plans conducted within *Fewer Falls in MS.* This assessment was based on follow-up telephone calls conducted after each reported fall to collect information about fall-related injuries and fall circumstances.

### Progression criteria

The research group established criteria (Table 1) to inform the decision on progressing to a full-scale RCT.

### Statistical analysis

Descriptive and comparative statistics were used to present the data. Normally distributed data were reported with mean, standard deviation (SD), and range; and non-normally distributed data with median and interquartile range (IQR). Categorical data were summarized using frequencies and percentages. Non-parametric statistics were applied for standardized questionnaire data. Between-group differences at T2 were evaluated using the Mann–Whitney U test for independent samples, except for questionnaires on physical activity and sedentary behaviour, where the Chi-square test was applied. Within-group differences were assessed using the Friedman test for related samples, except for questionnaires on physical activity and sedentary behaviour, where the Cochran’s Q test was used. Potential outliers were identified using box plots but were not removed from the analysis. Parametric statistics (independent t tests) were used to compare the number of falls, fall incidence (falls/person/year) and injurious fall incidence (injurious falls/person/year) between groups, with results presented as mean differences with corresponding 95% confidence intervals (CI). An intention-to-treat approach was adopted, utilizing all available data. All analyses were performed using SPSS statistics for Windows (release 29), with the significance level set at *p* < 0.05.

### Ethical approval

The study was approved by the Swedish Ethical Review Authority (registration number 2021–04817) and was registered at Clinical Trials before recruitment (Trial registration number NCT04317716). Procedures were conducted following the Declaration of Helsinki, and all trial participants provided written informed consent.

## Results

### Feasibility of the recruitment process

Recruitment and screening were conducted over 46 days, from January 11, 2022, to February 25, 2022. During this period, 63 interested individuals, from urban and rural areas across Sweden, contacted a research assistant and 56 (89%) were screened over the phone within the first five days. Fifty-seven (90%) reported receiving information about the trial through a newsletter from the patient organization Neuro Sweden. Of 61 eligible individuals, two declined participation and 11 did not return informed consent and/or did not complete baseline assessments. Thus, 46 participants, 38 ambulatory and eight non-ambulatory, were ultimately included in the trial (Fig. 1), representing 96% of the recruitment target. The recruitment resulted in 23 participants in each arm which was deemed sufficient to meet the study’s objectives.

### Feasibility of data collection procedures

The mean time needed for the 46 participants to complete baseline assessment was 14 min, ranging from 7 to 38 min. Forty-five participants completed the 7-week (T1) follow-up assessment, and 44 participants completed the 18-week (T2) follow-up assessment. Of the 44 participants who answered the evaluation questionnaire at T2, 42 (95%) strongly agreed that completing online questionnaires took a reasonable amount of time. Thirty-eight participants (86%) strongly preferred online questionnaires over paper versions.

Forty-five participants responded to the weekly SMS throughout the 18-week trial period. Forty-two responded to all weekly SMS and three participants missed one week each. A total of 107 telephone follow-ups, with some addressing multiple falls, were conducted by the research assistants. The telephone calls lasted approximately 10–20 min. Of the 45 participants monitored for falls, 32 (71%) participated in at least one telephone follow-up.

At T2, 44 participants, 39 (89%) strongly agreed that the SMS frequency was reasonable, and 38 (86%) agreed that receiving SMS on Saturdays was preferred over weekdays. Twenty-five (57%) strongly agreed that the telephone follow-up questions were easy to answer, and 27 (61%) strongly agreed that the follow-up took a reasonable amount of time. One participant strongly agreed that it was bothersome to be contacted by telephone.

### Feasibility of delivery of the intervention

Across the three groups, 19 of 21 online sessions were completed within the assigned time frame. Two sessions extended 10–20 min beyond the maximum allotted 2 h. There were some technical issues, primarily during the first three sessions, with some participants having difficulty joining the Zoom meetings and accessing their camera and/or microphone, and between sessions to log into the online learning platform. All participants could, however, participate in the sessions after receiving technical support. Despite these initial challenges, all planned sessions were successfully delivered.

### Feasibility of outcome measures

Data from the standardized questionnaires are presented in Table 3. None of the questionnaires had floor or ceiling effects (not shown in Table). Of 44 participants, 37 (84%) strongly agreed that the questionnaires were easy to understand, 35 (80%) that they were easy to complete, and 27 (61%) that they were relevant.

**Table 3:** Results from standardized questionnaires. Intervention group *n* = 23 at T0, *n* = 22 at T1, *n* = 21 at T2. Control group *n* = 23.

Outcome measure	Time	Intervention group		Control group		*P* value*
		Median	IQR	Median	IQR	
FPSS (0–22 points)	T0	11	8–12	11	8–12	
	T1	10	10–13	10	8–12	
	T2	10	9–14	10	8–15	0.93
*P* value^†^		0.96		0.48		
VAS-FoF indoors (0–100)	T0	42	20–50	30	20–64	
	T1	29	18–51	31	25–50	
	T2	25	13–50	30	17–69	0.54
*P* value^†^		0.23		0.65		
VAS-FoF outdoors (0–100)	T0	60	31–83	66	28–77	
	T1	55	38–77	64	30–75	
	T2	56	32–77	60	24–74	0.69
*P* value^†^		0.76		0.88		
FES-I (ambulatory participants) (16–64 points)	T0	33	27–42	34	22–40	
	T1	34	29–40	29	24–35	
	T2	34	27–38	31	23–39	0.69
*P* value^†^		0.66		0.28		
SCI-SCF (non-ambulatory participants) (16–64 points)	T0	40	26–54	39	28–51	
	T1	38	29–41	36	33–47	
	T2	34	30–	36	34–50	0.63
*P* value^†^		1.0		0.47		
FAI (0–45 points)	T0	25	18–33	29	23–32	
	T1	27	21–32	29	20–34	
	T2	29	22–37	29	21–34	0.81
*P* value^†^		0.03		0.27		
MSIS-29 physical (0–100 points)	T0	54	30–69	49	28–68	
	T1	51	31–65	46	25–58	
	T2	48	24–59	41	30–61	0.99
*P* value^†^		0.13		0.17		
MSIS-29 psychological (0–100 points)	T0	36	25–53	28	19–39	
	T1	35	24–45	31	19–42	
	T2	25	13–39	25	17–39	0.81
*P* value^†^		0.08		0.97		

		Intervention		Control		*P* value^*^
Physical activity < 150 activity min/week, n	T0	13		12		
	T1	10		10		
	T2	10		10		0.89
*P* value^†^		0.72		0.67		
Sedentary behaviour sitting ≥ 10 h/day, n	T0	14		9		
	T1	8		9		
	T2	9		9		0.80
*P* value^†^		0.10		1.0		

*FPSS* Fall Prevention Strategies Survey, *VAS-FoF* Visual Analogue Scale for Fear of Falling, *FES-I* Falls Efficacy Scale International, *SCI-SCF* Spinal Cord Injury Falls Concern Scale, *MSIS-29* Multiple Sclerosis Impact Scale, *FAI* Frenchay Activities Index. *Between groups, ^†^Within groups.

### Participant retention and session adherence

Of 46 participants, 42 remained in the trial. Three IGPs did not start the intervention due to personal reasons. One of these provided only baseline data and was considered a complete drop-out, the other two continued to participate in follow-up data collections at T1 and T2 and responded to the weekly SMS. One additional IGP was lost to the T2 data collection. There were no dropouts from the CG.

Ten of the 20 IGPs who started the intervention attended all 7 sessions and another eight attended 6 sessions.

### Between- and within-group differences

Results from the standardized questionnaires are presented in Table 3. There were no statistically significant between-group differences at T2. In addition, there were no statistically significant within-group differences except for FAI, where an increase in scores over time was observed for the IG.

**Fig. 3:**
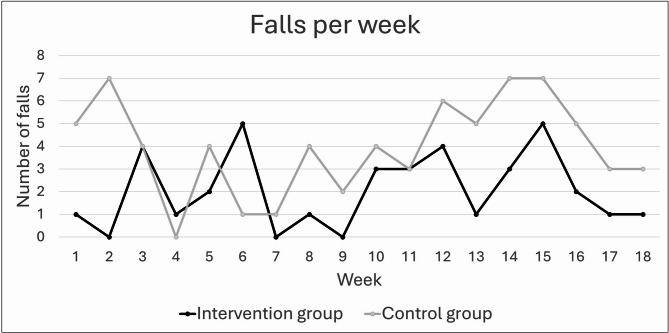
Number of falls per week in the intervention and control groups, respectively, during the 18-week data collection period.

Results from the collected falls data are presented in Table 4; Fig. 3. Fifteen ICGs reported in total 37 falls, and 17 CGPs reported in total 70 falls. There was a non-statistically significant trend for less falls among IG compared with CG (mean difference − 3.9, 95% CI − 8.9 to 1.1 *p* = 0.12), but with no difference in injurious fall incidence (mean difference 0.5, 95% CI − 1.6 to 2.6 *p* = 0.64).

**Table 4:** Results from the 18 weeks prospectively collected falls data.

	Intervention group (*n* = 22)	Control group (*n* = 23)
Number of fallers, n (%)	15 (68)	17 (74)
Frequent fallers (≥ 2 falls), n (%)	7 (32)	13 (57)
Number of falls, n	37	70
Fall incidence (falls/person/year), mean (95% CI)	4.9 (1.9–7.9)	8.8 (4.7–12.3)
Number of injurious fallers, n (%)	11 (50)	13 (57)
Number of injurious falls*	21	18
Minor	20	18
Moderate	0	0
Serious	1	0
Injurious fall incidence (injurious falls/person/year), mean (95% CI)	2.8 (1.1–4.4)	2.3 (0.9–3.7)

*Minor injury: minor bruises or abrasions not requiring health professional assistance; reduction in physical function for at least three days. Moderate injury: wounds, bruises, sprains, cuts requiring a medical/health professional examination such as physical examination, X-ray, suture. Serious injury: medically recorded fracture, head or internal injury requiring accident and emergency or inpatient treatment.

### Adverse events

There were no reported adverse events, i.e., fall-related injuries that participants perceived to be related to their participation in *Fewer Falls in MS*.

### Progression criteria

All predefined progression criteria except one were met (Table 1).

## Discussion

This parallel group pilot RCT demonstrated the overall feasibility of the *Fewer Falls in MS* intervention. Thus, the progression criteria for recruitment, data collection, intervention delivery, participant retention, and session adherence were all met (Table 1), validating a decision to proceed to a full-scale RCT to test the intervention’s effectiveness.

Participant recruitment was rapid and efficient, whereas previous fall prevention studies often report recruitment challenges^55^. The collaboration with Neuro Sweden played a key role, as most participants were recruited through their digital newsletter, highlighting the value of co-creation with patient organizations in clinical trials. The rapid recruitment also suggests a strong interest among PwMS in web-based fall prevention programmes, consistent with findings from a previous study^29^. Although no participant was excluded due to digital requirements, likely reflecting Sweden’s high level of digital literacy and widespread internet use^56^, the digital format may affect the generalizability of the findings. Individuals with limited digital access, a preference for in-person interaction, or impairments affecting communication may be less likely to participate in a fully online intervention.

The online format, combining real-time Zoom sessions with asynchronous content, enabled participation from individuals with higher disability levels, including non-ambulatory PwMS and those with fatigue, who are often underrepresented in similar studies^57^. Not having to travel and attend in-person sessions likely contributed to this inclusivity, as both travel and fatigue have been identified as barriers to participation in fall prevention interventions for individuals with neurological conditions^58^. Moreover, the online delivery allowed PwMS from across Sweden to participate, promoting equal access, especially for PwMS in rural areas where access to specialized care is often limited^59^. Despite this inclusive format, slightly fewer participants (*n* = 8, 17%) than the expected approximately 25%^60^ were classified as non-ambulatory. Ambulation level was based on self-reported walking ability, which may have led to an underestimation of the proportion of non-ambulatory PwMS.

The flexibility of remote delivery likely contributed to the high retention rate, with only one dropout and most participants completing follow-up assessments. Notably, two participants who did not start the intervention still engaged in follow-up assessments and weekly fall monitoring, indicating a strong commitment to the trial despite not receiving the full intervention. These findings highlight the potential of flexible online approaches to support retention in future trials.

Although the intervention was delivered as planned, there were some technical issues. This is in line with other MS fall prevention research that identified poor digital skills and limited computer access as barriers to participation^55^. Future trials may benefit from incorporating an initial technical orientation session to enhance participants’ confidence and reduce barriers to engagement. The group leaders received structured training to prepare for their role; however, future research is needed to explore how a train-the-trainer program could be designed to strengthen their confidence and further support their ability to create a supportive and engaging online environment while facilitating the *Fewer Falls in MS* intervention.

Data collection through online questionnaires and SMS was highly feasible, efficient and reduced the risk of missing data. Weekly SMS for fall monitoring minimized recall bias and offered a practical alternative to traditional falls diaries, which often suffers from poor return rates, lack of accuracy, and participant burden^24,26,27^. However, the follow-up telephone calls to collect data on fall-related injuries were time-consuming and would not be feasible in a larger trial. A self-administered online survey is recommended for future data collection on fall-related injuries. In line with our findings on the relevance of measures and recent recommendations for fall prevention outcomes in PwMS^61^, modifications on measures will be made. For example, the VAS-FoF will be replaced by the single-item question “Are you afraid of falling?” in future studies.

This pilot RCT was not powered to detect statistically significant changes between or within groups. While IGPs reported fewer falls than CGPs at follow up, there were some differences in the same direction also at baseline, which may have influenced the results. Moreover, given the short duration of the study, meaningful changes in fall outcomes were not to be expected. These findings underscore the need for a fully powered RCT with a larger sample size and longer follow-up period, to enable subgroup analyses and evaluate the programme’s effectiveness in reducing falls through sustained behavioural change. Maintaining behavioural change is a key challenge in fall prevention for PwMS, as initial improvements may decline over time^29^. To address this, *Fewer Falls in MS* includes components based on Social Cognitive Theory that target motivation and maintenance of action plans. Pilot results confirm the feasibility of the intervention, and the collected falls data will inform the sample size calculations for the full-scale RCT, which is designed to assess the programme’s long-term effectiveness in reducing falls over a 12-month period.

## Strengths and limitations

This pilot RCT, adhering to the Medical Research Council framework on complex interventions^37^, had several strengths. The RCT design and robust data collection methods, including online questionnaires, weekly SMS, telephone follow-ups, session observations, and the predefined progression criteria generated rich data for feasibility evaluation. A potential limitation was, however, that a history of falls was not an inclusion criterion, which may have reduced the relevance of the intervention for some participants. To address this, the full-scale RCT will include a history of falls as an inclusion criterion, in line with current recommendations^21^, ensuring the intervention is relevant and that peer learning is grounded in shared experiences. The relatively short follow-up period (18 weeks) is another limitation. While sufficient to assess short-term feasibility, it leaves uncertainties about long-term engagement, particularly with weekly SMS reporting. A planned one-year follow-up in the full-scale RCT will provide more robust data on engagement over time.

## Conclusion

Meeting all, but one (relevance of outcome measures), progression criteria justify proceeding to a full-scale RCT. Insights from this pilot trial will be used to further refine the intervention. To minimize technical difficulties in the future trial, an initial orientation session allowing participants to familiarize with the technology will be considered. Additionally, some modifications to outcome measures will be considered to increase relevance to PwMS.

## Supplementary Information

Below is the link to the electronic supplementary material.

Supplementary Material 1

## Data Availability

The datasets generated and/or analysed during the current study are not publicly available but can be available upon reasonable request. As data can indirectly be traced back to the study participants, according to the Swedish and EU personal data sharing legislation, access can only be granted upon request. Request for access to the data can be put to our Research Data Office (rdo@ki.se) Karolinska Institutet and will be handled according to the relevant legislation. In most cases, this will require a data processing agreement or similar with the recipient of the data.
