# Supplementary material for: Feasibility and outcome of the fewer falls in multiple sclerosis intervention: a pilot randomized controlled trial
Source: Sci Rep. 2025 Nov 18;15:40350. doi: 10.1038/s41598-025-27071-0 (PMC12627722; doi:10.1038/s41598-025-27071-0)
Supplement: Supplementary file 1 — Supplementary Material 1 [file 41598_2025_27071_MOESM1_ESM.pdf]

Evaluation of Questionnaires and SMS

The following questions are about your experience filling out questionnaires and reporting fall incidents.

Mark the answer that best matches your experience.

	1) Strongly agree	2) Partially agree	3) Do not agree at all	4) Not applicable
The questionnaire questions were generally easy to understand and answer.	◯	◯	◯	◯
It took a reasonable amount of time to complete the questionnaires.	◯	◯	◯	◯
The questionnaire questions felt relevant to me.	◯	◯	◯	◯
I prefer digital questionnaires over paper ones.	◯	◯	◯	◯
Receiving SMS once a week was frequent enough.	◯	◯	◯	◯
It was convenient for me to receive SMS on a Saturday.	◯	◯	◯	◯
I would have preferred to receive SMS on a weekday.	◯	◯	◯	◯
The interview questions after a fall incident were easy to answer.	◯	◯	◯	◯
The interview after a fall incident took a reasonable amount of time.	◯	◯	◯	◯
It was inconvenient to be called after a fall incident.	◯	◯	◯	◯
It was helpful for me to talk about my fall incidents.	◯	◯	◯	◯
Answering SMS and questionnaires made me think more about my fall risks.	◯	◯	◯	◯

Here you can add any additional comments about the questionnaires and SMS:
